# Oropharyngeal Bleeding Due to Cannabidiol Oil Vape Use

**DOI:** 10.7759/cureus.12676

**Published:** 2021-01-13

**Authors:** Rahul Pankhania, Alison Liu, Rob Grounds

**Affiliations:** 1 Ear, Nose & Throat, Royal Sussex County Hospital, Brighton, GBR

**Keywords:** bleeding, oropharynx, vape, oral injury, smoking, emergency medicine, ent, otolaryngology

## Abstract

Vaping has become an increasingly popular alternative to smoking in recent years. We present a rare and unusual case of upper airway bleeding caused by inhalation of a cannabidiol (CBD) oil-based vape due to a chemical burn. There are no case reports of this injury in the literature, and we discuss the clinical presentation, diagnosis and our management of this potentially life-threatening injury.

A 27-year-old man presented to the accident and emergency department after using a CBD oil vape. After one inhalation of the CBD oil vape, the patient experienced immediate onset pain in the oropharynx, dyspnoea, expectoration of blood and hoarseness. The patient had used a CBD oil vape four hours earlier that evening for the first time, which was procured from an unregulated online source.

The patient was referred to the Ear, Nose and Throat (ENT) team where the examination of oropharynx identified a posterior pharyngeal bleeding point. Flexible nasal endoscopy was undertaken showing profound erythema and inflammation throughout the oropharynx and posterior pharyngeal wall. The mucous membranes had been detached leaving an exposed bleeding submucosa.

The patient was commenced on three cycles of back-to-back adrenaline nebulisers (1:1000 adrenaline in 5ml of 0.9% NaCl), 6.6mg dexamethasone intravenously and hydrogen peroxide gargles (5ml of 3% hydrogen peroxide in 10ml of water) three times a day. There were early involvement and review of the airway by the anaesthetic and intensive care teams, which was deemed safe at the time. A plan was made for a definitive airway if bleeding reoccurred.

Upper airway bleeding can present as a rare form of vape-induced injury and should be considered part of the differential diagnosis particularly in those using CBD oil vapes. History taking is pertinent and patients should be questioned on the specific vape liquids used. Airway stabilisation is the priority with early involvement of the multi-disciplinary team including anaesthetists, intensive care specialists and ENT surgeons.

## Introduction

A 'vape' is a battery-powered device that generates an aerosol by heating a liquid that is emitted into steam and subsequently orally inhaled [1]. This process is often seen as a non-combustible alternative to smoking and is available in the form of several devices commonly electronic cigarettes, vapes and vape mods [2]. Liquids are often sold in cartridges or bottles and cannabinoid-containing liquids in the form of cannabidiol (CBD) oil, butane hash oils and tetrahydrocannabinol (THC) oils are commonly inhaled [3].

Vape usage has become an increasingly popular alternative to smoking in the past year particularly in a younger demographic of 18-30 year olds. Rare airway complications due to vape inhalation are poorly understood posing a serious diagnostic challenge. We present an exceptional case of sudden onset upper airway bleeding in a 27-year-old gentleman caused by CBD oil-based vape use. There are no case reports of this injury in the literature and we discuss the clinical presentation, diagnosis and our management of this life-threatening injury.

## Case presentation

A 27-year-old man presented to the accident and emergency department with a four-hour history of dyspnoea, expectoration of blood and a painful oropharynx. The patient had no history of trauma, no past medical history and no use of any regular medication. The patient had used a CBD oil vape earlier that evening for the first time which was procured from an unregulated online source. After one inhalation the patient experienced an immediate onset of their presenting symptoms. The patient was referred to the Ear, Nose and Throat (ENT) team where the examination of oropharynx identified a posterior pharyngeal wall bleed. Flexible nasal endoscopy was undertaken showing profound erythema and inflammation throughout the oropharynx and posterior pharyngeal wall. Mucous membranes were detached leaving an exposed bleeding submucosa.

The patient was haemodynamically stable on arrival and a baseline set of bloods and clotting studies were carried out, which were unremarkable (haemoglobin 156g/L, white cell count 10.9 x 10^9^/L, platelets 172 x 10^9^/L, C-reactive protein < 1 and international normalised ratio 1.1). A chest x-ray revealed clear lung fields throughout with no evidence of acute lung injury or lower respiratory pathology.

The patient was commenced on three cycles of back-to-back adrenaline nebulisers (1:1000 adrenaline in 5ml of 0.9% NaCl), 6.6mg dexamethasone intravenously and hydrogen peroxide gargles (5ml of 3% hydrogen peroxide in 10ml of water). These immediate measures stemmed the bleeding. The airway was reviewed by the anaesthetic and intensive care teams, which was deemed safe at the time and a shared decision plan with involvement from the multi-disciplinary teams was made for a definitive airway if bleeding reoccurred.

The patient was transferred for observation overnight to the high dependency unit and continued receiving humidified oxygen, hydrogen peroxide gargles and steroids. There were no further bleeding episodes. The patient was able to eat and drink the following day and subsequently discharged. At three-month follow-up, there were no further episodes of bleeding or sequelae. As a result of the incident, the patient had quit vaping.

## Discussion

Vape usage has grown exponentially over the past decade with approximately 3.6 million people in the UK (7.1% of the population) now vaping, a 12.5% increase from the previous year (2018). Despite the increasing use, there remains a large amount of uncertainty (27% of the UK adult population) as to how harmful vaping is compared to smoking [4].

There is extensive literature regarding vaping associated lung injuries affecting the lower respiratory tract, predominantly in the United States, which has seen a total of 2,807 hospitalised cases and 68 deaths as a result of vape usage [5,6]. These patients commonly present with dyspnoea, hypoxia, chest pain and diagnosis is confirmed on CT showing diffuse ground-glass opacities in the lungs [5]. There are no other case reports with a similar presentation to our clinical case. However, there are several case reports detailing CBD oil vaping causing acute lung injury and respiratory distress [7-11].

Vape-associated lung injury is strongly linked to the production of toxic compounds in the vape solution, in particular vitamin E acetate, which is used as a dilution agent and found in high concentrations of CBD oil solutions. The presence of toxic solvents is particularly specific to the CBD oil extraction process and often additives, such as propylene glycol, are used in the extraction process [12,13]. Propylene glycol when heated can transform this solvent into formaldehyde, which when ingested orally can cause lung injury [13,14]. Furthermore, vape-flavouring components, such as diacetyl, may also contribute to aldehyde production and affect respiratory function [15].

The combination of these caustic agents is often found in higher concentrations in unregulated vape CBD oil solutions. When heated and inhaled they can result in erosion of the mucosal surfaces causing bleeding [16]. Evidence surrounding the management of corrosive ingested substances is variable and is predominantly dependant on a case-by-case basis. Literature suggests removing the causative substance, ensuring the adequacy of the patient’s airway as a priority and avoidance of trying to neutralise the caustic material [16]. If the airway is unstable, a definitive airway should be carried out under direct visualisation.

## Conclusions

Upper airway bleeding can present as a rare form of vape induced injury and should be considered part of the differential diagnosis especially in vape users. As vape usage increases in popularity, this presentation may become more common, particularly to the emergency department. Thorough history taking is pertinent and specific details of vape liquids should be obtained. Clinicians should be aware of the appropriate management and early recognition of the potential life-threatening nature of this injury. Airway stabilisation is the priority with early involvement of the multi-disciplinary team including anaesthetists, intensive care specialists and ENT surgeons.
